# Reproductive Ecology of the Giant African Snail in South Florida: Implications for Eradication Programs

**DOI:** 10.1371/journal.pone.0165408

**Published:** 2016-11-18

**Authors:** Amy Roda, Gösta Nachman, Scott Weihman, Mary Yong Cong, Fredrick Zimmerman

**Affiliations:** 1 Miami Laboratory, Center for Plant Health Science and Technology, Plant Protection and Quarantine, Animal Plant Health Inspection Service, United States Department of Agriculture USDA-APHIS, Miami, Florida, United States of America; 2 Section of Ecology and Evolution, Department of Biology, University of Copenhagen, Copenhagen, Denmark; 3 Giant African Land Snail Eradication Program Miami, Division of Plant Industry, Florida Department of Agriculture and Consumer Services, Miami, Florida, United States of America; 4 Miami Plant Inspection Station, Plant Protection and Quarantine, Animal Plant Health Inspection Service, United States Department of Agriculture, Miami, Florida, United States of America; University of Minnesota, UNITED STATES

## Abstract

Giant African snail (*Achatina fulica* (Bowdich, 1822)), an important invasive snail, was recently found in South Florida, USA. An extensive eradication effort was initiated consisting of pesticide applications, debris removal and hand collections. We studied the reproduction capacity and population dynamics of snails collected from 22 populations for two years to help evaluate the likely success of the eradication program. A total of 23,890 snails, ranging from 25–131 mm, were measured, dissected and the number of eggs in each snail counted. Gravid snails ranged from 48–128 mm. Only 5% of snails had eggs, which were found year round. As the snails increased in size, they were more likely to include reproducing individuals. However, the percentage of gravid snails peaked when snails were approximately 90 mm. Although more prevalent, small (<65 mm) adults contributed fewer eggs to the population than the larger snails. We evaluated the effect of control measures on six populations having >1000 adult snails and used data from the two largest populations to investigate how environmental factors (temperature, humidity, and rainfall) interacted with population dynamics and control measures. More snails were collected in weeks with high humidity and more gravid snails were collected when the temperature was higher. The addition of metaldehyde pesticides had the greatest impact on population dynamics by reducing snail numbers. In populations with fewer snails, their numbers were already declining before the use of metaldehyde, although the new treatment accelerated the process. As a consequence of the eradication program, egg-producing snails were no longer collected from most populations by the end of the study. The aggressive and persistent control efforts apparently lead to reduced populations of egg producing snails, eventually resulting in local extinctions of this important pest.

## Introduction

Globally, the giant African snail (*Achatina fulica* (Bowdich, 1822)), is considered one of the most important invasive snails. It feeds on > 500 different plants, vector plants and animal pathogens, and may pose a threat to native flora and fauna [1, 2]. In addition, the snail presents a public health hazard due to its ability to spread diseases such as angiostrongylosis and eosinophilic meningoencephalitis while functioning as a host in the life cycle of *Angiostrongylus cantonensis* [2, 3]. The economic impact of the pest not only includes the monetary damage to crops but also costs for control measures.

Giant African snails (GAS) have spread from continental Africa to much of the tropical and subtropical world [4]. Commerce and intentional spread appear to be the most likely pathways for introduction of this pest to new areas [5, 6]. Once introduced, extensive and costly eradication efforts are often undertaken. Eradication efforts have been successful in the USA, Australia, and a few Pacific Islands in situations where the populations were small [2, 7, 8]. However, more often, the eradication effort is abandoned due to the rapid expansion of the pest range and the cost limitations of controlling the populations.

The cost of successful eradication has ranged from $60,000 for one seven-month effort [9] to $700,000 [USD in 1969 dollars] for a 10 year program in South Florida [5]. Naturally, the expense of eradication must be balanced against the estimated losses of leaving the pest unchecked. In the case of the 1960’s South Florida invasion, estimated losses would have been $11 million [USD in 1969 dollars] annually, well justifying the expense for a large eradication effort [5].

The extremely high reproductive capacity of GAS is one explanation for the snail’s invasiveness. Studies have shown that a 6 month-old snail is capable of laying 100 eggs, with the number of eggs increasing in subsequent years from 200–1800 [1, 10]. Population modelling indicates that under ideal growth conditions, 100 hatchlings were theoretically capable of producing a population in excess of 1000 individuals within 270 days [10]. With the snails living 3–5 years on average and the survivorship of eggs near 90%, a small introduction of snails can potentially grow to very high densities in a matter of years [11]. For example, millions of snails were collected during the first months of an eradication program in Guam [12], and biomass up to 780 kg ha^-1^ were reported in New Caledonia [13].

Potentially, a snail can lay a batch of eggs every few weeks as long as favorable conditions prevail [11]. However, studies have shown that the frequency of oviposition in the field rarely approaches this level [1]. In New Guinea, snails typically produce two clutches of eggs each year corresponding to the start of rainy seasons [14] and on Oahu, Hawaii 5–6 clutches of eggs are produced yearly [15]. In India, where snail activity is restricted to 4 months, snails produced 2–4 clutches in a study that followed reproductively mature snails for 4 years [10]. Environmental constraints may limit GAS population growth to a level that, with extensive control efforts, could help make eradication efforts successful.

In areas of the most recent invasion of the pest in tropical and subtropical North, Central and South America, countries have been undertaking efforts to eradicate GAS [16, 17]. Lush tropical foliage, abundance of calcium carbonate, lack of predators, warm climates, and areas of human-disturbed habitat are factors that strongly promote the survival, rapid population growth and dispersal of the pest [3, 7, 18, 19]. However, sub-tropical conditions, with dry seasons with low humidity and periodic freezes, may constrain population growth and expansion. In addition, the effects of climate change may increase the risk of invasion [20]. Understanding how the populations respond to conditions imposed by the new geographical location may help governments decide whether to attempt eradication in new areas of invasion.

In this study, we explore the reproduction potential of snails collected from the recently established populations in South Florida. Giant African snails were found in Miami-Dade County, Florida in October 2011 and the detection was immediately followed by the establishment of federal quarantine areas. An eradication program was quickly deployed focusing on debris removal to remove refuges, hand collection, and pesticide application [17]. The hand collection of a large number of snails provided an opportunity to determine the size and the egg production rate of GAS under fluctuating sub-tropical conditions of Miami-Dade County, FL. We also explored the effect of the control measures used in the eradication effort and discuss how understanding GAS reproductive capacity could be used to design more effective eradication strategies.

## Materials and Methods

### Field Locations and Snail Collection

**Fig 1:**
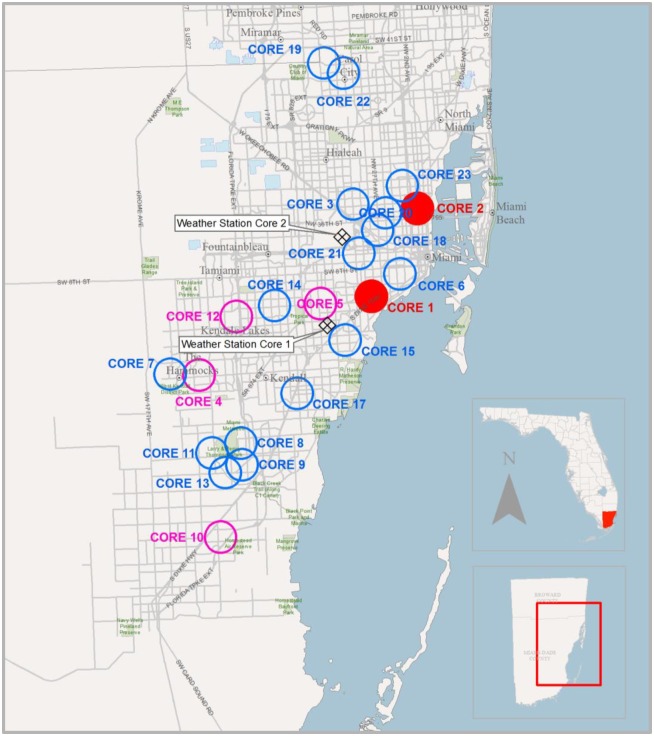
Populations (cores) of Giant African Snail located in Miami-Dade County, Florida USA, studied from March 29, 2012 through April 1, 2014.

Giant African snails were hand collected from over 600 residential properties located in Miami-Dade County, Florida from March 7, 2012 –April 1, 2014. The properties were located in one of 22 quarantine zones, termed cores (Fig 1). These were established by the United States Department of Agriculture (USDA) and Florida Department of Agriculture and Consumer Services Giant African Land Snail joint eradication program. Property owners within the cores signed release forms granting full permission for accessing and conducting control efforts. There were no endangered or protected gastropod species on the quarantine properties. The eradication effort entailed visual surveys, hand collection, debris removal, and pesticide treatments at least once every two weeks and only GAS were collected. All live and dead GAS found during the visual surveys were put into zip lock plastic bags, which were then placed in sealed buckets and transported to the laboratory. The date of collection and the property address were recorded. Snails collected from properties that had been treated with molluscicides were differentiated from newly discovered, untreated properties. The properties were designated “old” if they had undergone control measures including pesticides and “new” if they were from a recently discovered property and had not yet received pesticide treatment. In the laboratory, the number of live and dead snails were counted and sorted. The live snails were placed in a freezer for at least 24 hours then stored in 50% isopropyl alcohol until dissection.

### Snail Selection and Dissection

Due to the size of the infestation at the different cores and individual properties, the availability of snails varied greatly. Up to 100 snails, ranging in size from 25 to over 100 mm were collected weekly from each core when available for subsequent dissection in the laboratory. Previous observations (Zimmerman, unpublished data) showed that eggs could be present when snails reached a size of approximately 35 mm. Therefore, by choosing a minimum size of 25 mm it should be possible to estimate the smallest size at which eggs can be found in the oviducts. Digital calipers were used to measure shell length from the apex (pointed tip) of the shell to the furthest distance from the apex on the “lip” of the shell. The body of the snail was then extracted from the shell using forceps so that internal structures remained intact. If undamaged during extraction, the lung cavity was cut away to expose internal reproductive structures including any eggs that may be present. Individuals with fully developed eggs were characterized as being gravid. All fully formed eggs were counted. In addition, each egg was broken open to determine whether the embryo had developed into the stage at which the embryonic shell was visible. The numbers of eggs with and without an embryonic shell were recorded.

### Pesticide Treatments

All properties determined positive for GAS received calendar pesticide applications. From October 2011 through October 2012 pesticide applications were limited to 1% iron phosphate granular bait (Sluggo^™^) applied every two weeks. From October 2012 through April 2013, 5% boric acid (Niban^®^) was also used but limited to monthly applications due the low field efficacy [17]. Late March 2013, the eradication program received authorization from the USDA, Animal Plant Health Inspection Service to incorporate metaldehyde pesticides into the program at the manufacture recommended rates [21]. This included the use of 3.25% metaldehyde granules (Ortho^®^ Bug-Geta^®^) applied at a rate of 0.45 kg per 1341 m^2^ and 37.5% metaldehyde granules (Durham^®^) at a rate of 0.91 kg per 4047 m^2^. In addition, a liquid formulation of 25% metaldehyde (Slug Fest^®^) mixed at a rate of 20 ml per 7.5 l of water per 60 m^2^ were applied to properties with high densities of snails. From April 2013 and onwards, all properties with snails were treated with metaldehyde.

### Environmental Conditions

To determine the influence of environmental conditions on the number of snails collected and on their egg production, weather conditions were collected for Cores 1 and 2. These cores had the highest number of snails sampled (>6,000 snails) and the snails were present consistently throughout the 2 years of study. The fraction moon illumination at midnight was obtained from the Astronomical Applications Department of the U.S. Naval Observatory [22]. The temperature, rainfall, and humidity for each collection date were obtained from public weather stations using the daily rainfall and the minimum, maximum and average values of temperature and humidity recorded in South Miami, FL (25.714; -80.295) for Core 1 and at Miami International Airport, Miami, FL (25.795; -80.277) for Core 2 (Fig 1).

### Statistical Analyses

All statistical analyses were carried out by means of SAS Enterprise Guide 6.1 [23] and *P-*values less than 5% were considered as statistically significant.

#### Population trends

To investigate how the total number of collected snails (*N*), the total number of reproductive individuals (*R*), the average size of snails (*S*) and the average number of eggs per reproductive snail (*E*) varied from 25 March, 2012 through 30 March 2014, weekly data were fitted by means of multiple regression models using number of weeks from the first sampling date (*W*) and higher order terms of *W* as independent variables. Dependent variables showing variance heterogeneity were log transformed prior to the analyses. The statistical models were fitted to data by means of PROC GLM. The weekly variation in the proportion of snails that were reproductive (*p*) was analyzed by means of logistic regression (PROC GENMOD) applying the logit transformation (*y =* ln(*p/*(1-*p*))) as the link function and with deviance scaled for over-dispersion. The models generated the expected values of the dependent variables together with the 95% confidence intervals for the predicted lines.

#### Relationship between snail size and egg numbers

As larger snails may contain more eggs than smaller individuals [24], we explored the relationship between the size of a snail (*S*) and the number of eggs (*E*) in its oviduct by means of the equation

$$E=a{\left(S-S_{0}\right)}^{b},$$ (1)

where *S*_0_ is the minimum size at which snails start producing eggs, and *a* and *b* two shape parameters. Eq 1 has great generality as *b* = 0 implies that egg load is independent of snail size, while *b* = 1 means that *E* increases proportionally with *S-S*_0_. Furthermore, the relationship is concave for 0 < *b* < 1 and exponential for *b* > 1. The parameters were estimated by means of non-linear regression using PROC NLIN to fit the above model to egg counts obtained from 733 gravid snails.

#### Size class distribution and relative contribution of size classes to reproduction

The relationship between size and the number of eggs found in a snail was further explored to evaluate the potential contribution that snails of a given size would have made to the population. All the collected snails were grouped in 5 mm size classes, starting with the smallest gravid snail found in this study. Thus, snails < 45 mm were excluded from the analysis. In the model, *S*_*i*_ denotes the mid-point of the *i*th size class. The number of snails belonging to the *i*th size class was denoted *n*_*i*_ of which a proportion (*p*_*i*_) were gravid with an average egg load of *E*_*i*_ eggs per snail. The percentage of all snails in the *i*th size class was calculated as (*n*_*i*_/Σ*n*_*i*_)100%, while the percentage of all eggs in the size class was found as (*n*_*i*_*p*_*i*_*E*_*i*_/Σ*n*_*i*_*p*_*i*_*E*_*i*_)100%, where the sums were taken over all size classes for which *n*_*i*_ > 0. We used logistic regression (PROC GENMOD) to predict *p*_*i*_ as a function of *S*_*i*_ and Eq 1 to find the expected value of *E*_*i*_, i.e., *E*_*i*_ = *a*(*S*_*i*_*−S*_0_)^*b*^.

#### Factors affecting the abundance of snails

The population dynamics of snails in the large cores were analysed separately in order to identify factors that may have contributed to the changes in abundance over time. Each of the analysed cores had over 3500 snails collected and where sampled more than 40 times for a period of at least 18 months. This limited the analysis to 6 cores (1, 2, 4, 5, 10 and 12). We were especially interested in testing whether the populations declined over time and to what extent the decline could be attributed to pesticide application. We therefore omitted snails collected prior to the application of the pesticides. We further limited the analysis to snails capable of reproducing. Thus, snails < 45 mm were excluded from the analysis. Treatments were divided into two categories depending on whether the pesticide treatment involved metaldehyde (MH treatment) or not (Standard treatment). Standard treatment included hand collection, debris removal and the use of non-metaldehyde pesticides (iron sulphate and boric acid). The basic statistical model, based on a general linear model, included the class variable *Treatment* (Standard or MH), the covariate *W* (number of weeks since 25 March 2012) and the interaction between the two variables, i.e. Treatment*W. Environmental factors such as daily temperature (minimum, average and maximum), humidity (minimum, average and maximum), rainfall, and percent moon illumination were averaged on a weekly basis and used as covariates in the general linear models in order to investigate whether any of these variables could account for the observed deviations from the general trends in snail abundance. As the environmental variables were measured only for Core 1 and 2, the analysis was limited to these two cores. PROC GLMSELECT was used to identify the best model, based on a stepwise selection procedure and with Schwarz Information Criterion [25] as selection criterion. All parameter values of the final model were significantly different from 0.

#### Factors affecting the proportion of adult snails

We investigated whether the proportion of reproductive individuals out of all snails ≥ 45 mm sampled in Core 1 and 2 could be related to the same factors as used in the previous analysis. PROC GENMOD was used to fit the logistic model to the observed values of the dependent variable *p =* reproductive snails/all snails. To find the best model, non-significant terms were gradually omitted from the full model until all remaining terms were significantly different from 0.

## Results

### Population trends

In total 23,890 snails were collected from 22 cores and dissected (Table 1). The number of snails available that met the size criterion (*i*.*e*. ≥ 25 mm) varied greatly between the different locations and was related to the size of the snail population and to the number of samples. Some cores such as number 1, 2, 10 and 12 had larger snail populations and snails were collected on more of the sampling dates (Table 1). Other cores such as number 3, 6 and 7 had fewer snails and sample dates. During the course of the study 9 new cores (Cores 14–23) were discovered and included in the analysis. These additional areas, all located in Miami-Dade Co., occurred less than 20 km from the other cores and were found in similar suburban (non-agriculture) habitat (Fig 1). Also during the course of the study, live snails were no longer or only sporadically being collected from most of the older, original cores. The addition of the new cores allowed continued monitoring of reproductive individuals experiencing the impacts of both the eradication program and the environmental factors of Miami-Dade County.

**Table 1:** The dates and number of giant African snails collected from 22 locations (cores) located in Miami Dade County, Florida, USA.

Core*	Date of Start Sampling	Date Last Sample	# Weeks with Snails**	Total # Dissected	# Snails > 47.5 mm	# Snails with Eggs	% Reproductive***	Total # of Eggs	Mean # Eggs
1	3/30/12	12/5/13	75	3482	2672	206	7.7%	15792	76.7
2	3/27/12	1/24/14	89	3962	2819	138	4.9%	11747	85.1
3	4/21/12	7/30/13	15	32	27	2	7.4%	182	91.0
4	4/14/12	8/9/13	49	1670	862	20	2.3%	2460	123.0
5	3/27/12	6/27/13	49	1745	1204	72	6.0%	6371	88.5
6	12/19/12	1/10/14	10	82	53	10	18.9%	961	96.1
7	7/5/12	4/13/13	7	13	11	2	18.2%	457	228.5
8	3/7/12	3/11/14	45	1320	746	28	3.8%	3335	119.1
9	3/30/12	3/5/14	48	678	270	12	4.4%	864	72.0
10	3/22/12	2/19/14	73	4078	2315	117	5.1%	10864	92.9
11	3/31/12	11/26/13	36	794	375	12	3.2%	1913	159.4
12	4/11/12	4/4/14	84	3567	2249	81	3.6%	9310	114.9
13	4/17/12	12/23/13	16	82	48	2	4.2%	156	78.0
14	5/9/12	8/10/13	18	51	45	0	0.0%	0	0.0
15	5/25/12	3/9/13	22	583	321	5	1.6%	496	99.2
17	8/4/12	5/4/13	14	265	111	1	0.9%	231	231.0
18	8/30/12	9/3/13	21	1184	356	13	3.7%	1087	83.6
19	11/7/12	11/20/12	2	12	9	1	11.1%	100	100.0
20	11/13/12	11/29/12	3	133	37	1	2.7%	111	111.0
21	5/7/13	11/13/13	5	61	55	12	21.8%	648	54.0
22	9/11/12	11/21/13	2	30	10	1	10.0%	93	93.0
23	12/6/13	2/14/14	2	10	4	1	25.0%	291	291.0

*Core 16 consisted of only one snail and was not included in the study

**Based on a total of 112 sample weeks with each Core surveyed and treated every other week

*** % reproduction based on the smallest size snail found with eggs (48 mm)

**Fig 2:**
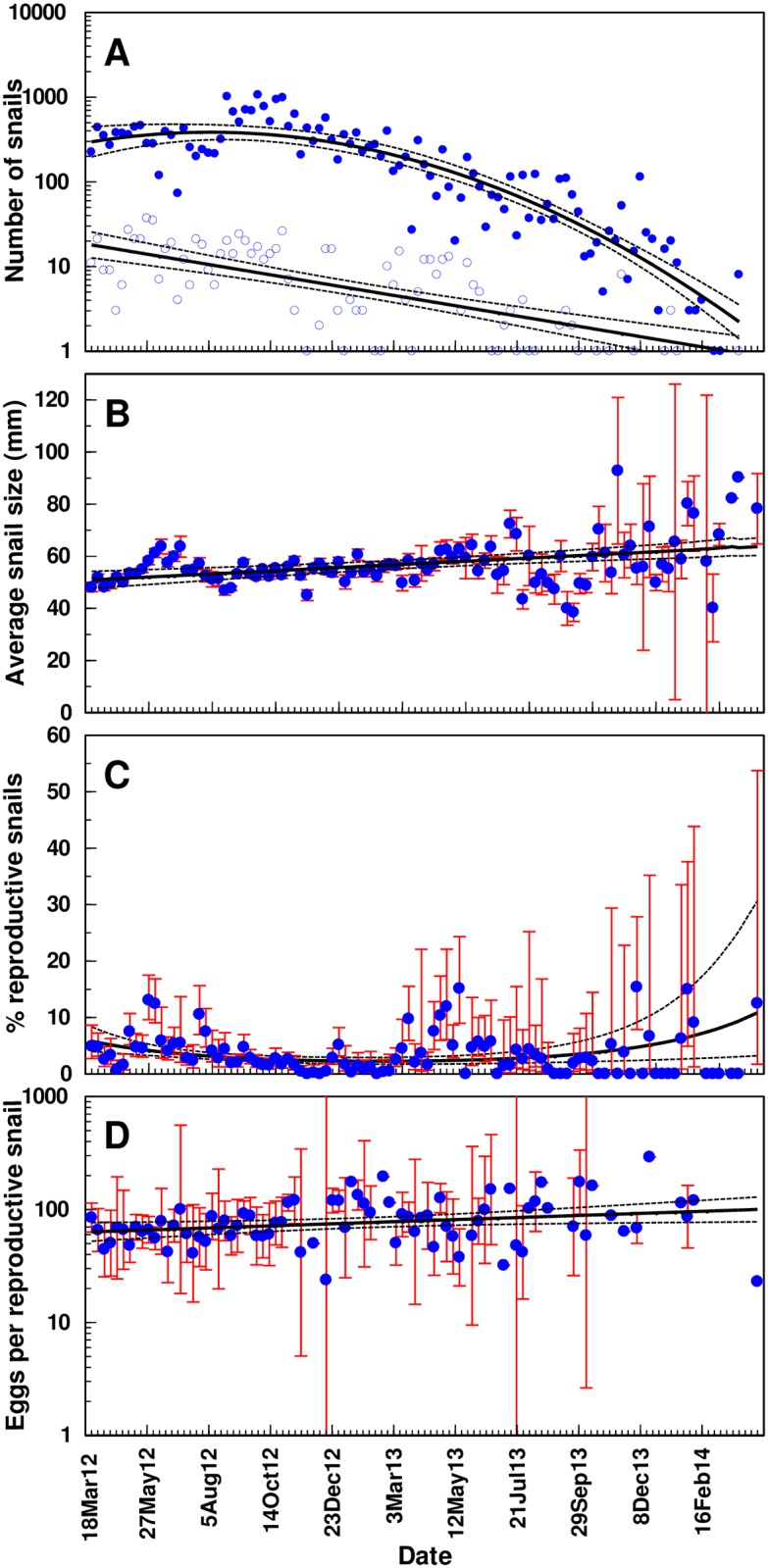
Population dynamics of giant African snails collected from 22 populations (cores) located in South Florida, U.S.A. from 25 March, 2012–30 March 2014. **A**: Number of snails (filled circles: all individuals; open circles: gravid individuals). **B**: Average shell size. **C:** Percentage of gravid snails. **D**: Average number of eggs per snail. Thin vertical lines show 95% confidence limits for mean values (only when *n* > 1). Trend lines with 95% confidence limits are shown.

There were more snails found without eggs than those with eggs throughout most of the study period. The model fit to the weekly numbers of snails was (with standard error of the estimated parameter values in parentheses) log *N =* 2.4726(0.0896) + 0.0117(0.0040) *W–* 0.00030(0.00004) *W*^2^, indicating that the snail population peaked in Miami-Dade Co. in August 2012 (Fig 2A). The model explained 80.5% of the variation in the empirical values of log *N* (PROC GLM: *R*^2^ = 0.805; *F*_2,99_ = 204.8; *P <* 0.0001). There was a steady decline in the number of snails with eggs over the two years (Fig 2A) as the best model fitted to data was log *R =* 1.2553(0.0766) - 0.0122(0.0015)*W* (PROC GLM: *R*^2^ = 0.454; *F*_1,78_ = 64.87; *P <* 0.0001). The difference between the number of snails with eggs and the total number of snails became negligible by late winter and early spring of 2014 as the overall availability of live snails from the 22 cores was greatly reduced. The average size of the dissected snails (*S*) increased steadily throughout the study (Fig 2B) with a trend line given as *S* = 50.642(1.610) + 0.1205(0.0267) *W* (PROC GLM: *R*^2^ = 0.165; *F*_1,103_ = 20.43; *P <* 0.0001). Beginning in spring of 2013 there was an increase in variability around the mean size, which corresponded with the decrease in snail abundance (Fig 2A).

The proportion of snails with eggs was low with an overall average of 5% for all cores over all sample dates and ranged between 0–25% (Table 1). The smaller cores, from which fewer snails were collected, showed the largest divergence from the mean percent reproduction (Table 1). The proportion of reproducing individuals showed an initial decrease then subsequently an increase over time. The trend line for the proportion of reproductive snails (Fig 2C) was obtained from *p* = ln(*e*^*y*^/(1+*e*^*y*^)), where *y =* ln(*p*/(1-*p*)) *=* -2.6211(0.2252)– 0.0473(0.0126)*W* + 0.0005(0.0001)*W*^2^ which explained 13.3% of the deviance of the null model (PROC GENMOD: *F*_2,736_ = 56.47; *P <* 0.0001). The confidence limits around the model predicted line increased from spring 2013 as there were fewer snails available.

Though snail abundance initially showed an increasing trend followed by a persistent decline, this long-term pattern was seemingly overlaid by a seasonal variation, which was most apparent in snails collected prior to the wide scale use of metaldehyde in the eradication program (Fig 2). A general pattern occurred where from spring through early fall a larger percentage of snails had eggs (average of 8% ranging from 4–16%) followed by a drop in late fall through winter (average of 2.5% ranging from 0–7%). The percentage of snails with eggs increased again the following spring and summer (average of 7% ranging from 2–17%).

### Relationship between snail size and egg numbers

**Fig 3:**
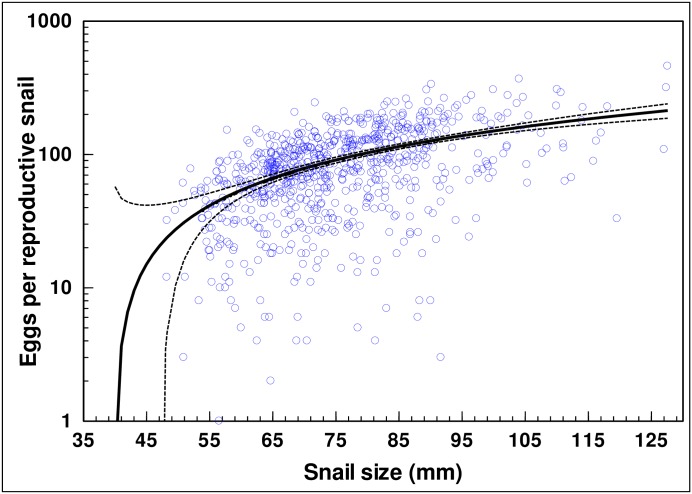
Relationship between the size of gravid giant African snails and the number of eggs produced per snail. Dots: Observed values. Full line: Predicted relationship. Broken lines: 95% confidence limits.

The mean number of eggs was 113 eggs/gravid snail and ranged from 1–460 eggs in individuals (Table 1). This average number of eggs found in snails did not change throughout the two year study although there was large variability found each week (Fig 2D). The size of a snail affected how many eggs were found in its reproductive tract (Fig 3). The smallest snail found to have eggs was 48.22 mm. However, most snails with eggs ranged from 65–100 mm with an overall mean of 75 ± 0.5mm. The parameters of Eq 1 were estimated as *a =* 3.2826 (SE = 3.2790), *b* = 0.9331 (SE = 0.2155) and *S*_0_ = 39.8834 (SE = 8.3727). The model explained 77.8% of the total variation in the observed number of eggs per snail which was highly significant (PROC NLIN: *F*_3,729_ = 853.8; *P <* 0.0001). The model predicts that the minimum size of reproductive snails (*i*.*e*. snails with at least one egg in the reproductive tract) is 40.16 mm but the confidence limits are seen to be very wide for snails of this size. As snails grew larger they produced more eggs, with the largest snail (128 mm) having the most (460) eggs. However, large snails (> 70mm) were also found at times to have fewer than 10 eggs. With these individuals the ovisac was often observed extended versus tightly covering the eggs, suggesting that the snail was collected before ovipositing the full complement of eggs.

### Size class distribution and relative contribution of size classes to reproduction

**Fig 4:**
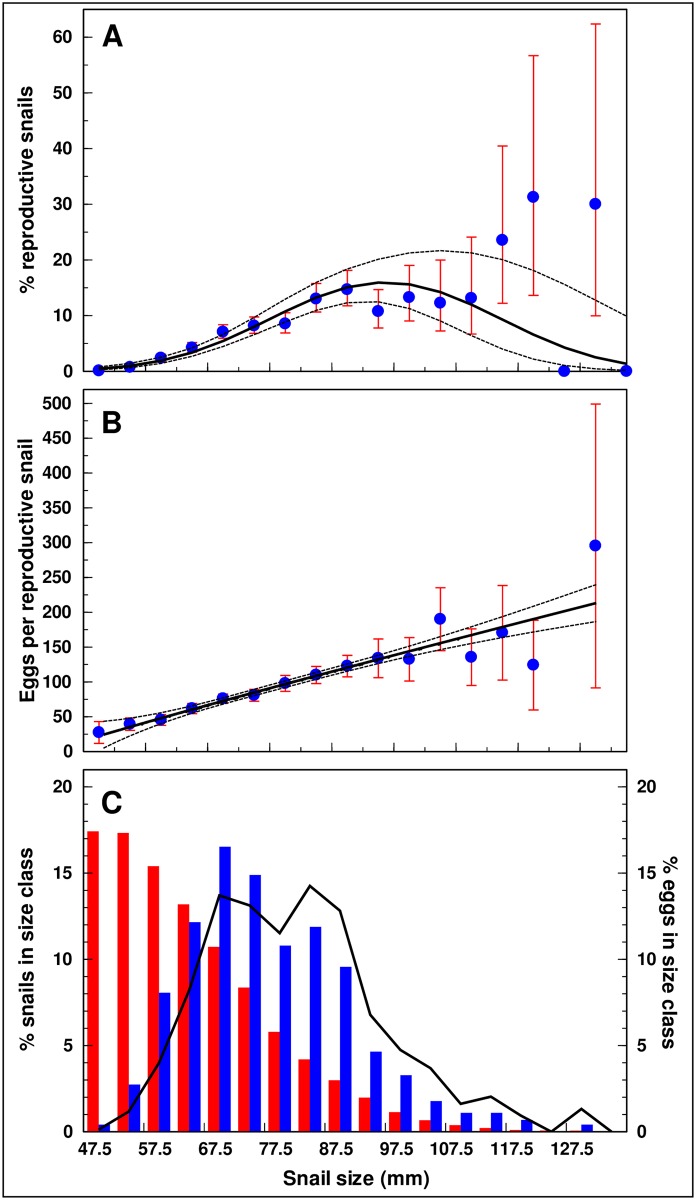
Relationship between the size of giant African snails and (A) the percentage of reproductive snails, (B) the number of eggs per reproductive snail, and (C) the distributions of snails (left axis) and eggs (right axis) per size class. Data are grouped in size classes of 5 mm. Dots and vertical lines: Observed values with 95% confidence limits for the mean (for points where *n >* 1). Full and broken lines: predicted values with 95% confidence limits. Bars: Percentage of all snails (red) and reproductive snails (blue) in size class *S*.

Although there were more snails in the smaller size classes there were fewer individuals with eggs and they contributed with fewer eggs relative to the larger snails (Fig 4A). The predicted relationship between size and the proportion of reproductive snails (*p*) was obtained as *y =* ln(*p*/(1-*p*)) = -17.2638(0.8493) + 0.3347(0.0226)*S–* 0.0018(0.0001)*S*^2^ (Fig 4A). The model indicates that as the snails increased in size they were more likely to include gravid individuals until they reached approximately 90 mm where the percentage of reproducing individuals in these large size classes levelled off and eventually declined. The predicted decrease was associated with considerable uncertainty due to the small number of collected snails. Thus, the model closely matched the South Florida observed values for all size classes except the largest snails (>110 mm). A higher percentage of the larger-sized field-collected snails were found with eggs than predicted by the model. The predicted relationship between snail size and the number of eggs produced per reproductive snail was based on Eq 1, using the midpoint of each size class as the predictor variable (Fig 4B). The predicted relationship closely matched the observed values for all size classes up to approximately 110 mm when the confidence limits became very broad due to the small number of large individuals.

While the majority of snails belonged to the smaller size classes, the highest percentage of reproductive individuals was observed when size was between 65 and 70 mm (Fig 4C size distributions of all snails (red bar) and reproductive snails (blue bar)). Although the percentage of egg-producing individuals declined with sizes above 70 mm, the number of eggs per individual continued to increase. As a result, snails belonging to the size class between 80 and 85 mm contributed the most to the total egg output (Fig 4C line). Overall, however, snails between 60 and 90 mm were most important for the reproductive capacity of the population.

### Factors affecting the abundance of snails

**Fig 5:**
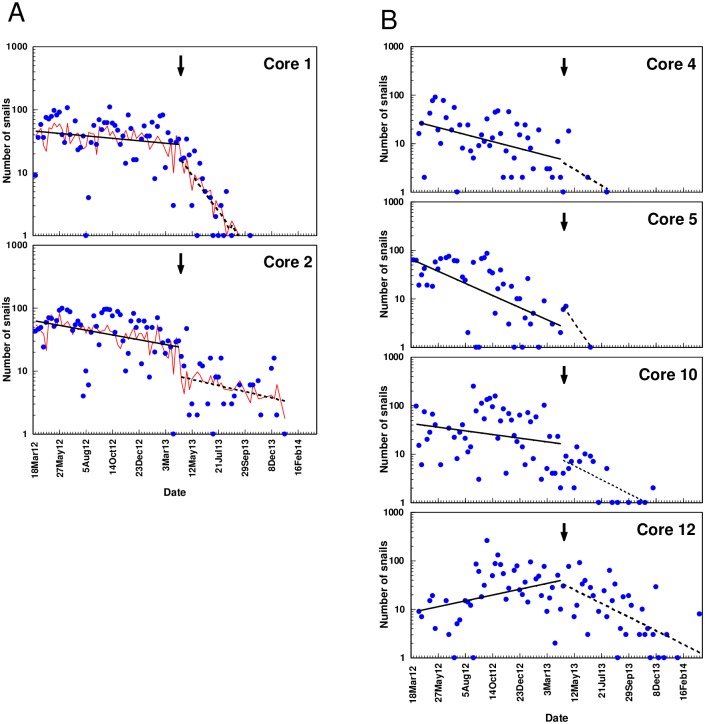
Factors affecting the abundance of snails from 25 March 2012 through 30 March 2014. A. Number of giant African snails (≥ 45 mm) in Core 1 and 2. B. Number of Giant African Snails (≥ 45 mm) in Core 4, 5, 10 and 12. Trend lines for the periods where snails were controlled by means of standard (full bold line) or metaldehyde (broken bold line) treatments are shown. The arrows mark the change from standard to metaldehyde treatments. Thin lines show the predicted number of snails using treatment, time and the average daily humidity (*mHumAvg*) for each week as predictor variables (Core 1 and 2 only).

Measures to control the populations of snails were found to affect the abundance of snails (≥ 45 mm) in the selected cores (Fig 5). The statistical model based on a general linear model that included the class variable *Treatment* (Standard or MH) and the covariate *W* (number of weeks since 25 March 2012), showed that the populations were declining during the Standard treatment (full bold lines) in all cores except Core 12, while all populations declined during the MH treatment (broken lines). The statistical analysis showed that the decline in snail abundance over time during the Standard treatment was significant in Core 1 (*P =* 0.05), Core 2 (*P =* 0.0079), Core 4 (*P =* 0.0027), Core 5 (*P* < 0.0001), and Core 10 (*P =* 0.0147). The decline in snail abundance was accelerated in all cores during use of MH, but the difference between slopes before and after the introduction of MH was only significant in Core 1 (*P =* 0.0002) and Core 12 (*P <* 0.0001).

When the environmental factors were included in the models predicting the snail dynamics in Core 1 and 2, the only variable that contributed significantly to reduce unexplained variation was the average humidity (*mHumAvg*). In both cores, the parameter (*β*) associated with this variable was positive, indicating that high relative humidity (% RH) during the week where individuals were sampled increased the number of snails being collected (Core 1: *β* = 0.0135(0.0044); *t*_68_ = 3.05; *P =* 0.0033. Core 2: *β =* 0.0183(0.0056); *t*_76_ = 3.24; *P =* 0.0018). Incorporating *mHumAvg* in the model increased the explained variation (*R*^2^) from 0.59 to 0.64 in Core 1 and from 0.54 to 0.60 in Core 2.

### Factors affecting the proportion of egg-producing snails and eggs with developed embryos

**Fig 6:**
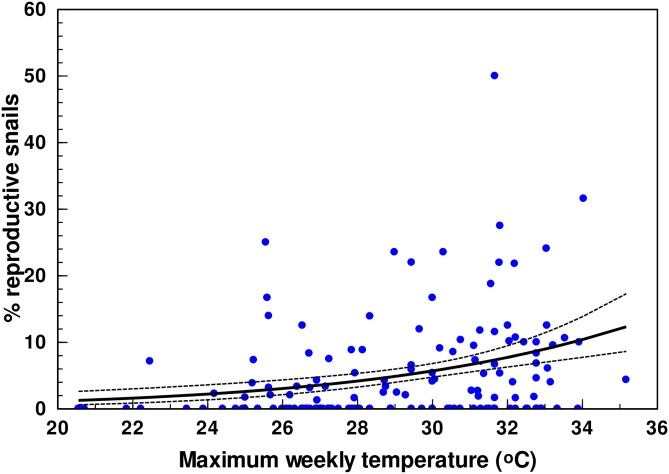
Percent of gravid snails occurring in Core1 and 2 each week from 25 March 2012 through 30 March 2014 plotted against the maximum weekly temperature (dots). The heavy full line shows the predicted relationship based on logistic regression with 95% confidence limits (broken lines).

The proportion of gravid snails was found to be independent of time and treatment in both Core 1 and 2. We therefore omitted these factors from the model and lumped data from the two cores. The best model to predict the proportion of reproductive individuals was found as *y =* ln(*p*/(1-*p*)) = -10.6023(1.7103)+0.0907(0.0197)*mTempMax*, indicating that snails with eggs constituted a larger part of the sampled individuals in weeks with high maximum temperatures (*mTempMax*), though with a considerable scatter (Fig 6). The model explained 13.4% of the deviance of the null model (PROC GENMOD: *F*_1,150_ = 21.20; *P <* 0.0001). When the same analysis as the one described above was carried out on the proportion of snails (≥ 45 mm) containing embryos with developed shell, we found the best model to fit data as *y =* ln(*p*/(1-*p*)) = 3.0167(2.6326)– 0.1050(0.0312)*mHumMax*, which explained 16.3% of the null model’s deviance (PROC GENMOD: *F*_1,150_ = 8.92; *P* = 0.0028). As *mHumMax* denotes the weekly average of the daily maximum humidity, the model indicates that the proportion of snails collected that had eggs with developing shells was highest during dry weeks. Snails with shelled embryos were rare being present in only 8 of the 153 samplings.

## Discussion

Program decision makers must determine if resources should be directed toward eradication of an invasive pest or if those resources would be better directed toward developing management strategies for the pest. Eradication programs for GAS have been frequently started upon discovery of the species in a new area [5, 16, 17]. However many eradication programs are abandoned or are not even considered, particularly if the targeted populations are large and dispersed over a wide area [1, 3, 12, 26]. The reproductive capacity of individual GAS is often given as a defining reason why the snails escaped control efforts and eradication programs had to be abandoned. Determining how effective eradication strategies are at removing snails that otherwise could produce eggs may help decision makers recognize the likelihood of success as they would learn at what level individuals are continuing to contribute to the future population. This study showed that factors in the reproductive biology of field collected South Florida GAS both improves and poses challenges for the likelihood of eradication.

Snails in the range of 60–90 mm were most important for the reproductive capacity of the South Florida population. The statistical model developed from the two years of data showed that as the snails increased in size they were more likely to include gravid individuals. At approximately 90 mm, the percentage of gravid individuals levelled off and eventually declined. Concurrent with the declining percentage of egg-producing individuals, the number of eggs per reproducing individual continued to increase. As a consequence, snails sized 80–85 mm contributed the most to the total reproductive output. In addition, snail survival has been found to be highest in large clutches. In a laboratory study, clutches numbering less than 75 eggs were associated with poor hatching ratios and high rates of post-hatching mortality [27]. Thus removing large snails (> 60 mm) from the population is critical to reducing the reproductive output of the pest and may thereby contribute to the success of an eradication program.

The eradication strategy taken in South Florida appears to be effective at removing the reproducing snails. The snail population peaked in August 2012 and was followed by a steady decline over the two year study. A closer analysis of the largest cores showed that this trend was significantly increased after the initiation of metaldehyde treatments. A similar pattern was observed in the two smaller cores (Core 4 and 5), though the regression slopes before and after addition of metaldehyde could not be shown to be significantly different. The high efficacy of metaldehyde is confirmed by laboratory and caged field experiments where very high lethality rates (> 80%) were achieved while alternative pesticides with active ingredients such as methiocarb and iron phosphate show only moderate efficacy (< 50% mortality) [9, 17, 28]. In addition, the average snail size continued to increase throughout the study period while the total number of snails declined (Fig 2), which suggests that the smaller snails were more vulnerable to the treatments as has been found in laboratory studies [17]. Without recruitment of younger snails, the old and large individuals will gradually dominate a declining population.

This study showed that overall only a very small proportion of the potential population had eggs at a given time. In the cores with the largest number of adult snails (>1000 individuals per core) this percentage ranged from 2–8%. This is a great benefit to the eradication effort when viewed that 92–98% of the reproductive sized snails that could be producing an average of 113 eggs are not contributing these new individuals to the population. A similar low percentage was found on Hahajima Island, Japan, where only 2.2, 1.3 and 5.3% collected in 1995, 1998 and 2001, respectively, had eggs [29]. However, the percentage of gravid snails can be greater. For example, 20% of the snails collected from three sites on Chichijima Island, Japan, had eggs [24] and on Oahu, Hawaii, an 18 month study showed egg-laying to be cyclical and at the peak of reproduction 14 to 15% of the population had eggs [15]. A similar pattern was also found in South Florida where more snails (up to 14%) were found with eggs during the warmer, humid and rainy months of May through mid-October.

In South Florida, snails with eggs were found every month of the year including the cooler and drier winter months. This is in contrast to other field studies where snail activity and egg production have been found to decline during drier months [10, 11, 14, 30]. The snails often completely stop producing clutches of eggs and, depending on the severity of the conditions, enter prolonged periods of estivation lasting from 2–10 months [1, 10, 30]. Histological studies of snails collected in Bangkok, Thailand, showed that, although oocytes were found the entire year, the number of oocytes per acinus was lowest in March, which corresponded to conditions of low humidity, high temperature and limited vegetation availability [31]. The presence of snails with eggs during the dry and cooler months may have resulted from sporadic showers or possibly be attributed to microclimatic effects created by the urban environment such as lawn irrigation, ornamental ponds, plants that catch water and condensation from air conditioners. The foraging activity of snails during the cooler and drier winter months has been observed to be greater on properties with these conditions (Roda and Yong Cong unpublished data). In addition, Achatinidae are able to retain eggs [1]. While eggs may be deposited within 8–20 days of mating [30, 32], egg production was observed over 350 days after mating [10]. Egg retention over prolonged periods of estivation may provide the snails with the capability to produce eggs at any time of the year given favorable environmental conditions [1]. More eggs with embryo development were found in snails during the winter months further supporting the idea that the South Florida snails are seasonally ovoviviparous. For the eradication effort, this study showed that adult snails should be continuously targeted in both dry and rainy seasons. Logically, the threat of pest expansion is greatest when more snails are producing eggs. However, the presence of gravid snails with developed embryos during the dry season warrants concerted effort to remove them prior to the onset of favorable conditions.

In South Florida, we found that snails measuring 48–128 mm were capable of producing eggs, which included snails both smaller and larger than reported in the literature. Studies conducted on Chichijima Island, Japan showed intermediate (52.2±10.1 mm) and old snails (61.2±8.34 mm) had eggs [24]. Likewise, Lange [26] found that snails from Saipan, ranging from 57 to 88 mm had eggs, while studies in Hawaii showed that snails (66–112 mm) found copulating in the field produced eggs while being held in the laboratory [15]. For the South Florida eradication program, the presence of gravid snails both smaller and larger than this range could pose problems. Smaller snails reach egg laying size possibly within or sooner than a year [10, 27] and are more likely to escape visual detection. Larger snails cause a greater risk as they frequently had more eggs than smaller snails as seen in this and other studies [24, 32]. The study in South Florida also showed that the largest snails continued to produce eggs contrary to what was reported in previous studies [1, 27, 33]. Pawson and Chase 1984 showed that snail fecundity of laboratory colonies peaked between the age of 210 and 270 days followed by a marked decline with almost no clutches produced by animals older than 1 year. A similar pattern was found in the field, although the time to reach maximum egg production and the rate of subsequent decline corresponded with the slower growth rates under field conditions [1] and egg production stopped several months after reaching full sexual maturity [14, 33]. In contrast, the largest snails (>112 mm) from South Florida not only had eggs but the largest clutches were found in some of these individuals.

Although the aggressive and persistent eradication efforts significantly reduced the number of egg producing snails leading to their apparent local extinction, the South Florida program continues to face the challenge of reaching complete eradication. The use of metaldehyde-based pesticides clearly remains one of the more important tools, however, alternative control strategies are essential to reach eradication. Mortality rates of GAS to metaldehyde have been shown to be dose-dependent with higher percentage of active ingredients needed to cause death with larger snails [28]. In a laboratory study where snails were repeatedly treated with low, sub-lethal doses of metaldehyde, the mortality rate declined over time [28]. The snails’ feeding behavior also affects the efficacy of the pesticides. When allowed to avoid the baits in choice test studies, the efficacy of pesticide baits dramatically decreased compared to no choice assays where the snails had only the bait present [17]. In addition, large snails do not tend to range far from an established location, often returning to the same home location each night [34]. In a South Florida residential environment, the snails persist in protected areas such as areas between property fences, or are concealed in thick brush or debris (Roda and Yong Cong unpublished data). Recent reoccurrences of snails in the original cores are often from these types of habitats that prevent access for hand collection or pesticide treatments. A single large snail escaping detection could potentially reestablish a growing population.

The risk that only a few snails can lead to a major infestation was seen in 1966 when GAS established in 9 residential areas in South Florida as a result of the introduction of only 3 juvenile snails carried from Hawaii [7]. After 7 years and treatment of 25,680 properties, the species was finally declared eradicated [7]. Thus, eradication, or at least temporary suppression of the species below non-detectable levels in a widespread area seems possible provided adequate financial and human resources remain available for multiple years. As the management of invasive species has become an increasingly important problem, understanding the factors of the pest’s biology that are likely to effect the success of a control effort is critical. This study of the reproductive ecology of GAS while under an active eradication program may be helpful in developing efficient control strategies that will prevent GAS from becoming a serious pest particularly in new areas of invasion.

## Supporting Information

S1 Data The size of snails, the number of eggs found after dissection and the environmental conditions occurring the time of collection. (XLSX)
